# Taxonomic notes on the genus *Orthobrachia* Warren, with description of a new species from China and Thailand (Lepidoptera, Geometridae)

**DOI:** 10.3897/zookeys.609.8288

**Published:** 2016-08-08

**Authors:** Guo-Hua Huang, Zi-You Su, Dieter Stüning

**Affiliations:** 1 Hunan Provincial Key Laboratory for Biology and Control of Plant Diseases and Insect Pests, Hunan Agricultural University, Changsha, Hunan 410128, P. R. China; 2 Sichuan Forestry Inventory and Planning Institute, Chengdu, Sichuan 610081, P. R. China; 3 Zoological Research Museum Alexander Koenig, Adenauerallee 160, D-53113 Bonn, Germany

**Keywords:** Ennominae, Orthobrachia hirowatarii sp. n., Sichuan province, taxonomy

## Abstract

All seven members of the genus *Orthobrachia* Warren, 1895 are recorded, with description of a new species from Sichuan Province, China and N. Thailand, including *Orthobrachia latifasciata* (Moore, 1888) and *Orthobrachia flavidior* (Hampson, 1898) from northern India, Nepal and China, *Orthobrachia tenebrosa* Yazaki, 1992 from Nepal and India, *Orthobrachia owadai* Yazaki, 1992 from India, *Orthobrachia simpliciata* Yazaki, 2002 from China, and *Orthobrachia maoershanensis* Huang, Xin & Wang, 2003 from South China. A key to the *Orthobrachia* species is provided, along with a distributional map of all nominal species. The type specimens of the new species are deposited in Hunan Agricultural University (China), South China Agricultural University (China) and Zoological Research Museum Alexander Koenig (Germany).

## Introduction

The genus *Orthobrachia*,﻿ belonging to the subfamily Ennominae (Geometroidea: Geometridae), was established by Warren in 1895 with *Stegania latifasciata* Moore, 1888 as its type species. The genus remained monotypic until the same author one year later described *Orthobrachia particolor* Warren, 1896 from the Khasi Hills, India. However, this species later was transferred to *Crypsicometa* Warren, 1894 by Prout (1915), without any further comments —neither on the genus *Orthobrachia* nor its type-species *latifasciata* Moore — in this work. Wehrli (1939) treated *Orthobrachia* as a subgenus of *Lomographa* Hübner, [1825] sensu auct. (= *Stegania* Guenée, [1845]), and Inoue (1987) placed its type-species *latifasciata* into *Heterostegane* Hampson, 1893, thus synonymizing *Orthobrachia* with *Heterostegane*. Yazaki (1992) finally revived *Orthobrachia* as a valid genus (and described two new species), but based this treatment on comparison with the genera *Stegania* and *Heterostegane* (which belong to the tribe Cassymini) and not to more closely related genera like *Crypsicometa* Warren, 1894 or *Heterostegania* Warren, 1893 (which belong – like *Orthobrachia*,﻿ to our opinion – to the tribe Baptini). *Crypsicometa* was later synonymized with *Platycerota* Hampson, 1893 by Stüning 2000. Scoble (1999) also treated *Orthobrachia* as a valid genus and included four species. Subsequently, Yazaki (2002) and Huang et al. (2003) named further two new *Orthobrachia* species from Taiwan and Guangxi, respectively. To date, six species are reported worldwide (Scoble 1999; Yazaki 1992, 2002; Huang et al. 2003): *Orthobrachia latifasciata* (Moore, 1888) (type locality Darjeeling, India) and *Orthobrachia flavidior* (Hampson, 1898) (type locality Khasi Hills, India) from N. India, Nepal, Thailand, Vietnam and China, *Orthobrachia tenebrosa* Yazaki, 1992 (type locality Gandaki Parbat District, C. Nepal) from Nepal and India, *Orthobrachia owadai* Yazaki, 1992 (type locality West Sikkim) from India, *Orthobrachia simpliciata* Yazaki, 2002 (type locality Taiwan) from China, and *Orthobrachia maoershanensis* Huang, Xin & Wang, 2003 (type locality Guangxi) also from China.

Recently, some geometrid moths were collected at Longcanggou Town, Yingjing County, Sichuan Province, among them one species of the genus *Orthobrachia* which was confirmed as new to science and will be described herein. Surprisingly, one additional specimen of this species was discovered among material from N. Thailand in the ZFMK collection.

In the present paper, seven species from the Oriental Region are treated, and the adults and the genitalia are illustrated. Diagnostic generic characters of adults are proposed, and a key to all species is provided, based on external features. A distributional map of all *Orthobrachia* species is presented.

## Material and methods

Adults of the new species were collected by light trap. The types of previously described species, deposited in the Natural History Museum, London, UK (BMNH) and in the National Science Museum, Tokyo, Japan (NSMT), were examined. Other specimens examined in this study are deposited in South China Agricultural University
(SCAU), Hunan Agricultural University
(HUNAU), China and Zoological Research Museum Alexander Koenig, Bonn, Germany (ZFMK).

The methods for examining the genitalia and taking the photographs are as described in Wang et al. (2015). Morphological terminology in descriptions follows Kristensen (2003). Type specimens of the new species described here are deposited in HUNAU, SCAU and ZFMK.

## Taxonomy

### Orthobrachia

Taxon classificationAnimaliaLepidopteraGeometridae

Warren, 1895

Orthobrachia Warren, 1895. Novit. zool. 2: 121. Type species: Stegania latifasciata Moore, 1888 by original designation. In: Hewitson & Moore, Descr. new Ind. lep. Insects Colln late Mr. Atkinson: 260. [Type specimen: Lectotype, male, Darjeeling in India (BMNH, London), designation by Yazaki in Haruta, 1992]

#### Description and diagnosis.

Head. Male antennae shortly and stiffly bipectinate to three-fourths or four-fifth, rami unscaled on dorsal side, flattened and distally slightly clubbed, arising ventrally from about the middle or the proximal one third of the flagellomers. Female antennae filiform. Frons narrow, flat, smooth-scaled. Palps delicate, slightly curved upward, reaching just beyond frons. Basal segment rough-scaled, second segment smooth-scaled, terminal segment very small. Haustellum well developed. Thorax. Hind leg tibia not dilated, without scent-brush. Index of spurs 0-2-4. Forewings without fovea; common stalk of veins R_2_-R_5_ arising at large distance from the upper corner of the cell (= origin of M_1_) in forewing, very close to the origin of vein R_1_ which anastomoses with Sc. In hindwing vein Rs also at large distance from M_1_. Abdomen with tergites and sternites weak, membranous, only tergites 1 and 2 and sternites 1+2 may be slightly sclerotized. Sternite 3 without setal comb. Tympanal organs rather large, globular, without laciniae.

Characters indicating *Orthobrachia* to be a distinct genus are found in the male genitalia: Elongated, spined or densely setose lobes arising from dorsal margin of sacculus near its base are present in all species of *Orthobrachia*, but are not found in other genera of Baptini. The valves are elongated, more or less parallel-sided, with rounded apex, curved upward in all species except *Orthobrachia simpliciata*, with a broad, immaculate zone with one or two processes of different shape; valve lamina with an elongated field of setae; gnathos weak, only lateral arms present; juxta a broad, oval, somewhat elongate plate, ventrally angled and with v-shaped incision distally; aedeagus with bulbous caudal end, curved or straight, with one or two large cornuti on vesica, in two species with a bunch of external cornuti or a long row of small cornuti.

The female genitalia also indicate the distinctness of *Orthobrachia*, though their characters separate two species-groups: 1) the *latifasciata*-group, also containing *flavidior*, *tenebrosa* and *owadai*: their genitalia are characterized by a well sclerotized antrum with distal and/or lateral processes, continued into a long, sclerotized band that reaches deeply into the bursa copulatrix. Signum a large, ring-like structure without dentation; 2) the *simpliciata*-group with semicircular lamellae antevaginales, without long sclerotized bands and with a stellate signum. *Orthobrachia simpliciata* exhibits the most plesiomorphic characters in the female, but also in the male genitalia.

#### Host-plant.

Unknown.

#### Distribution.

**Figure 1.**
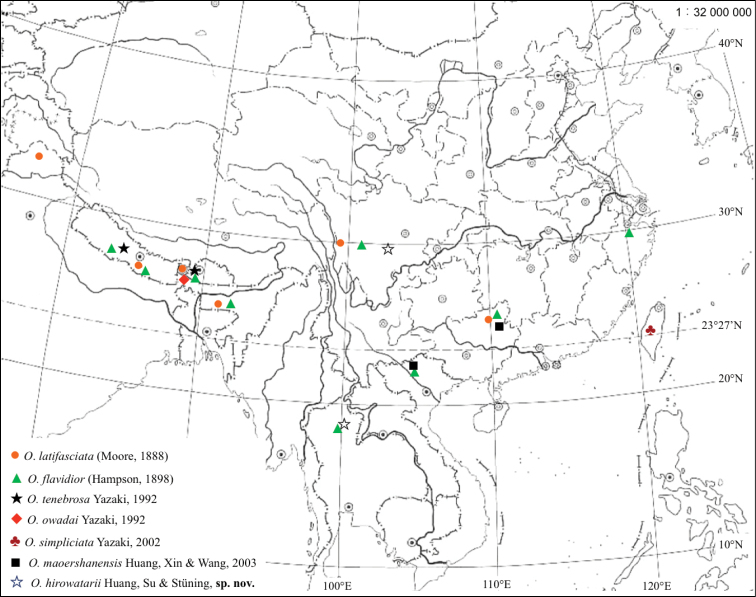
Distributional map of *Orthobrachia* species.

Oriental Region. The distributional map of all the known species is provided in Figure 1.

#### Key to the species of the genus *Orthobrachia*

(based on wing pattern and colouration)

**Table:** 

1	Hindwing pale yellow with incomplete postmedial line only, or with a large pale greyish-brown medial area	**2**
–	Hind wing orange-yellow or pale yellow, with a white or greyish-white medial band	**3**
2	Hind wing with incomplete postmedial line only	***Orthobrachia simpliciata***
–	Hind wing with a large, pale grey or grey-brown medial area	**5**
3	Medial band of hind wing white or greyish-white, postmedial area consisting of a greyish-brown apical part and a large, oval, orange-yellow or pale yellow posterior part. Transverse lines of forewings waved	***Orthobrachia latifasciata***
–	Medial band of hindwing clear white, postmedial area almost entirely orange-yellow or pale yellow	**4**
4	Transverse lines of forewing waved	***Orthobrachia owadai***
–	Postmedial line almost straight from costa to vein CuA_1_	***Orthobrachia flavidior***
5	Transverse lines of forewing waved, tornal and medial area greyish-brown, except for an oval, pale yellow patch near costa	***Orthobrachia tenebrosa***
–	Transverse lines of forewing straight or nearly straight, medial area predominantly pale yellow, only partly dark brown	**6**
6	Postmedial line slightly curved inward near costa, tornal dark brown patch reaching rather shortly into the medial area; dark grey postmedial line of hind wing evenly curved, reaching costa basally from apex	***Orthobrachia maoershanensis***
–	Postmedial line straight, tornal dark brown patch narrow, but almost reaching antemedial line; dark grey postmedial line of the hindwing straight or almost straight between apex and tornus	***Orthobrachia hirowatarii***

### Orthobrachia latifasciata

Taxon classificationAnimaliaLepidopteraGeometridae

(Moore, 1888)

**Figure 2.**
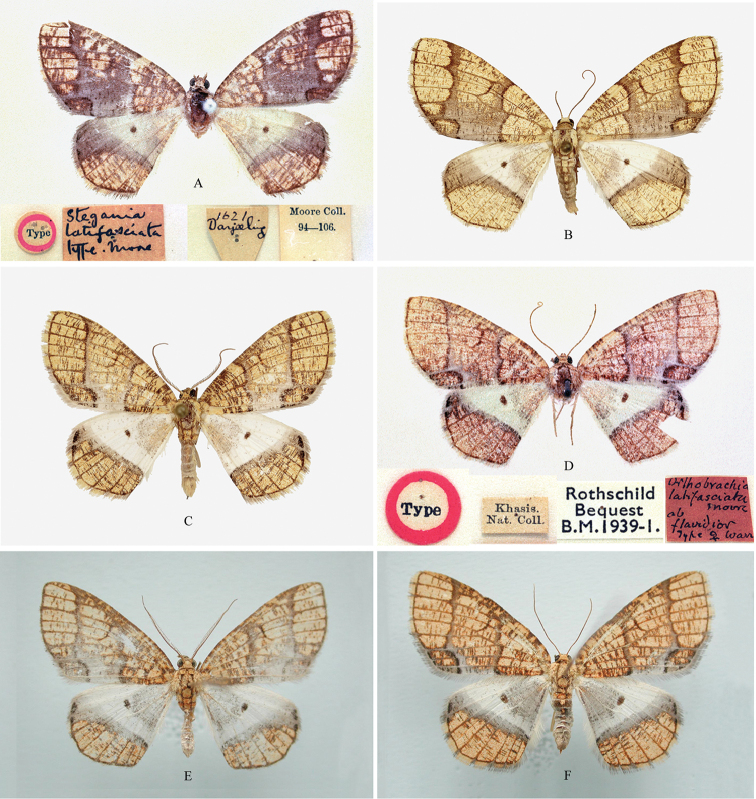
Adults of *Orthobrachia* species. **A–B** *Orthobrachia latifasciata* (Moore, 1888) **A** male from Darjeeling in India, lectotype **B** female from Nepal **C–F** *Orthobrachia flavidior* (Hampson, 1898) **C** male from India **D** female from India, Lectotype **E** male from Guangxi province in China **F** female from Guangxi province in China.

Figures 2A–B , 4A , 5A

Stegania latifasciata Moore, 1888, in Hewitson & Moore, Descr. new Indian lepid. Insects Colln late Mr Atkinson: 260. Type locality: Darjeeling, India.
Orthobrachia latifasciata : Yazaki 1992: 21.

#### Diagnosis.

This species can be recognized and distinguished from the other *Orthobrachia* species based on external features. Main diagnostic characters are the strongly waved antemedial and postmedial lines in forewing, the white or slightly greyish-white medial bands and the large, dark brown apical patch with concave posterior border in hind wing (see also key to species). In addition the genitalia of both sexes exhibit distinctive characters: valva with a large, dorsal saccular lobe, formed like a heterocercal fish-tail in a lateral view, broadly oval in dorsal view. Compared to *flavidior*, the uncus is more slender, and the shorter and broader valva of the latter has a large basal lobe instead of the hook-like costal process just beyond the middle of the valva. The aedeagus of *latifasciata* is long and slender, with one stout cornutus, that of *flavidior* is shorter and rather stout, with two cornuti. The female genitalia are distinctive, with three long spines distally on the antrum, while *flavidior* has two short distal and two long proximal spines laterally on antrum. A second similar species is *owadai*, also having a white median band in hind wing (diagnosis given under *owadai*).

#### Material examined.

INDIA: 1♂, Lectotype of *Stegania latifasciata* Moore, designated by Yazaki (1992), labeled “Type/ *Stegania latifasciata* Moore, Type/1621 Darjeeling/ Moore Coll. 94-106/ Geometridae genitalia slide No. 7925 ♂”, BMNH; l♂2♀♀, W. Bengal, Tiger Hill, 2573 m, 30.IX-5.X.1986, F. Aulombard & J. Plante leg., BMNH. l♂1♀, “Khasias”, L. B. Prout Coll., B.M. 1939-643 (ZFMK, by exchange from BMNH, 1964); NEPAL: l♂, Godavari, 28.VI.1990, preserved in BMNH; Gandaki Parbat District, 1♀, Ghorapani, Deolari, 2800 m, 15.X.1981, M. Owada leg., NSMT; 1♀, Ulleri, 2070 m, 14.X.1981, M. Owada leg., preserved in NSMT; 3♀♀, Banthanti, 2620 m, 16.X.1981, M. Owada leg., NSMT; l♂2♀♀, nr Kathmandu, Siwapuri 2650 m, 7.X.1981, M. Owada leg., NSMT. 1♂, Indien, Jammu & Kashmir, Ladakh, 15 km E Drass, 3000 m, 1.VIII.1986, leg. W. Thomas, Gen. prep. no. 2306-DS, ZFMK; CHINA: 1♂2♀♀, Tieshanting, Mao’ershan National Nature Reserve, 1950 m, 11.IX.2015, M. Wang leg., SCAU; 1♀, “Frontière orientale du Thibet, Chasseurs indigènes du P. Déjean 1906”/ *Lomographa*, *Orthobrachia* Warr., *latifasciata* Moore ♀, abgebildet Seitz IV. Suppl. fig. 22g, ex coll. Ch. Oberthür. ZFMK.

#### Biology.

The adults are flying in summer and autumn.

#### Distribution.

N. India, Nepal and China.

#### Remarks.

This species is distributed in the high mountains, usually above 2000 m and up to 3000 m in altitude.

### Orthobrachia flavidior

Taxon classificationAnimaliaLepidopteraGeometridae

(Hampson, 1898)

Figures 2C–F , 4B , 5B

Orthobrachia latifasciata ab. flavidior Warren, 1896, Novit. zool. 3: 128. Unavailable, infrasubspecific.
Stegania latifasciata var. flavidior Hampson, 1898, J. Bombay nat. Hist. Soc. 11: 714. Type locality: Khasi Hills, India.
Lomographa latifasciata flavidior Warren [sic]: Wehrli 1939: 296. Incorrect authorship.
Orthobrachia flavidior : Yazaki 1992: 22.

#### Diagnosis.

This species is very similar to *Orthobrachia latifasciata*, but is generally smaller and can be distinguished by external features and characters of the genitalia: the postmedial line of forewing is almost straight or just slightly curved inward from costa to tornus, the clear white medial bands of hind wing are narrowly bordered dark grey outside, but a large, dark apical patch is absent; the uncus is broader and a little shorter than in *latifasciata*;﻿ the valva is shorter and broader and has a large, roundish, sclerotized lobe at base of the costa and lacks a median, hook-like costal process found in *latifasciata*. The basal saccular process is much larger and longer and arises more distally. A second saccular lobe is missing. The aedeagus is rather stout, with a round apex, which is bluntly pointed in *latifasciata*. The cornuti, consisting of a pair of stout spines situated on a diverticulum of the vesica, are somewhat longer than in *latifasciata*. In the female genitalia, the antrum is well sclerotized, nearly quadrate, with two pairs of processes, longer at caudal and shorter at distal margin, while in *latifasciata* it bears three longer distal spines.

#### Material examined.

INDIA: 1♀, Lectotype of Orthobrachia latifasciata ab. flavidior Warren, designated by Yazaki (1992), labeled “Type/ Orthobrachia latifasciata Moore ab. flavidior Warr. Type ♀/ Khasis Nat. Coll. /Rothschild Bequest B. M. 1939-1/ Geometridae genitalia slide No. 15658 ♀”, BMNH; 1♀, Paralectotype of Orthobrachia latifasciata ab. flavidior Warren, “Khasis, May 1896, Nat. Coll.”/ Collectio H. J. Elwes/ ZFMK, by exchange from BMNH, 1964; l♂, Khasia Hills, Assam, Nissary; Joicey Bequest. Brit. Mus. 1934-120/ ZFMK, by exchange from BMNH, 1964; l♂, India, W. Bengal, 2400 m, Darjeeling, Tigerhill, 10-12.VII.1986, leg. W. Thomas. ZFMK; 1♀, same locality & collector, 29-31.VIII.1986, ZFMK. NEPAL: l♂, Godavari, 2.V.1990, preserved in BMNH; l♂, Mt. Phulchouki 21.VII.1990, BMNH. l♂, Pokhara, 2 km S Kharey, 1785 m, 21–25.II.2009, leg. T. Ihle & S. Löffler. ZFMK; l♂, Gandaki Kaski District, Naudanda, 1470 m, 12.X.1981, M. Owada leg., NSMT. CHINA: 1♀, Tieshanting, Mao’ershan National Nature Reserve, 1950 m, 11.IX.2015, M. Wang leg., SCAU. l♂, West-Tien-Mu-Shan, 1600 m, Pz. Chekiang, 18.VII.1932, H. Höne. ”/ *Lomographa latifasciata* [sic] Moore ♂, abgebildet Seitz IV. Suppl. Fig. 22g, ZFMK; 1♀, “Chasseurs Indigènes des Missionaires de Ta-tsien-Lou, 1906”, ex coll. Ch. Oberthür. ZFMK. THAILAND: l♂, Changwat Chiang Mai, Doi Phahompok, 18km NW of Fang, 2100 m, 7.II.2000, leg. Hreblay & Szabó, ZFMK; VIETNAM: l♂, N. Vietnam, Mt. Fan-si-pan, 2250 m, 1-6.XI.1995, leg. V. Sinjaev & E. Afonin; l♂1♀, N. Vietnam, Mt. Fan-si-pan, 1500-1800 m, 10.VI-6.VII.1994, leg. V. Sinjaev & local collectors (ZFMK).

#### Biology.

The adults are flying from February to November.

#### Distribution.

N. India, Nepal, Thailand, Vietnam and China.

#### Remarks.

This species was firstly described by Warren (1896) as *latifasciata* ab. *flavidior*, which is nomenclaturally unavailable. Hampson (1898) raised the name to a species-group rank, stating, “The Khasi form has the dark markings of forewing reduced, and the white band of hindwing broader.” — The “*latifasciata*”-male from Zhejiang figured in Seitz 4, Suppl. on plate 22, line g, is misidentified and in fact a male of *flavidior*. It is preserved in coll. ZFMK and could be checked by D.S.

### Orthobrachia owadai

Taxon classificationAnimaliaLepidopteraGeometridae

Yazaki, 1992

Figs 3B , 4D , 5D

Orthobrachia owadai Yazaki, 1992, Tinea, 13 (Suppl. 2): 23. Type locality: West Sikkim, India.

#### Diagnosis.

This species can be distinguished from other *Orthobrachia* species, especially from the very similar female of *latifasciata*, based predominantly on the following characters: In the forewing, the postmedial line is situated more distally and less deeply waved. In the hindwing, the postmedial line is shaded distally with brown, more broadly than in *flavidior*, ﻿but much less than in *latifasciata*. In the latter the whole apical one-third is dark brown, with a rounded posterior border. In the male genitalia, the valva has a large triangular, distally rounded costal process just before the middle, a second, smaller one at the base of the valva and the saccular lobes are shorter and broader, roundish, curved basad. The aedeagus is slender with a bunch of short spines externally near apex, the vesica bears two cornuti situated one after another on a large diverticulum (looking like one large cornutus in our Fig. 4D). In the female genitalia, a large lamella postvaginalis is absent and the antrum bears only two spines distally, pointing laterally. Longitudinal sclerotized band and round, ring-like signum are uniting *owadai* with the three species mentioned before.

**Figure 3.**
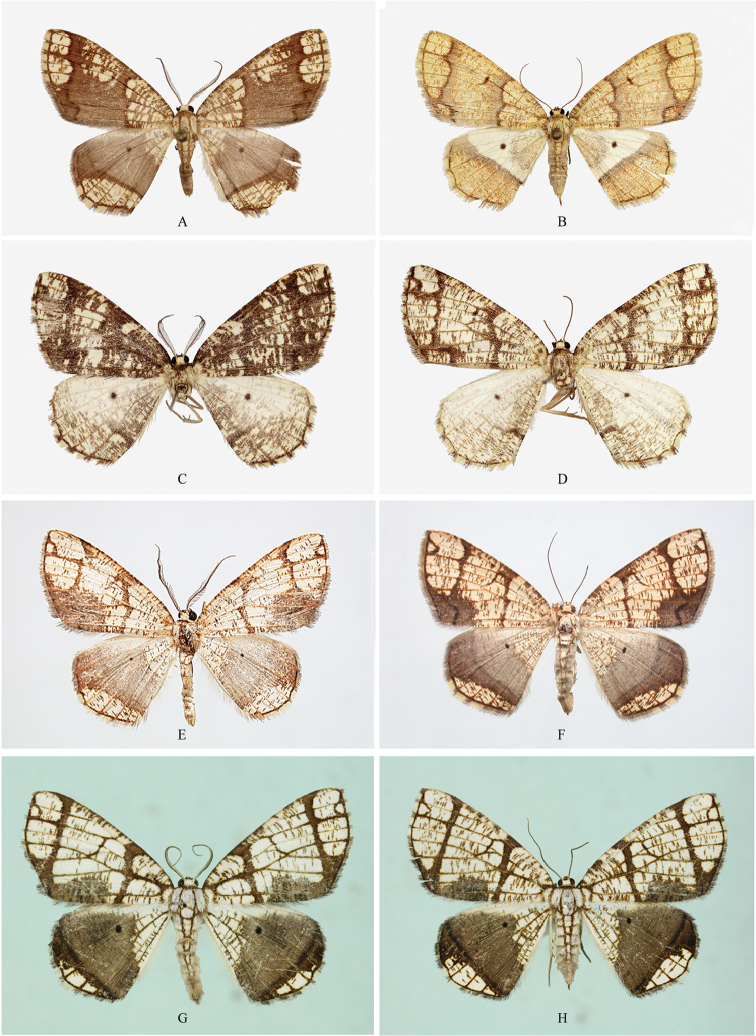
Adults of *Orthobrachia* species. **A** *Orthobrachia tenebrosa* Yazaki, 1992, male from Nepal, paratype **B** *Orthobrachia owadai* Yazaki, 1992, female from Nepal, paratype **C–D** *Orthobrachia simpliciata* Yazaki, 2002 **C** male from China, paratype **D** female from China, paratype **E–F** *Orthobrachia maoershanensis* Huang, Xin & Wang, 2003 **E** male from Guangxi Province in China, holotype **F** female from Guangxi Province in China, paratype **G–H** *Orthobrachia hirowatarii* Huang, Su & Stüning, sp. n. **G** male from Sichuan Province in China, holotype **H** female from Sichuan Province in China, paratype.

**Figure 4.**
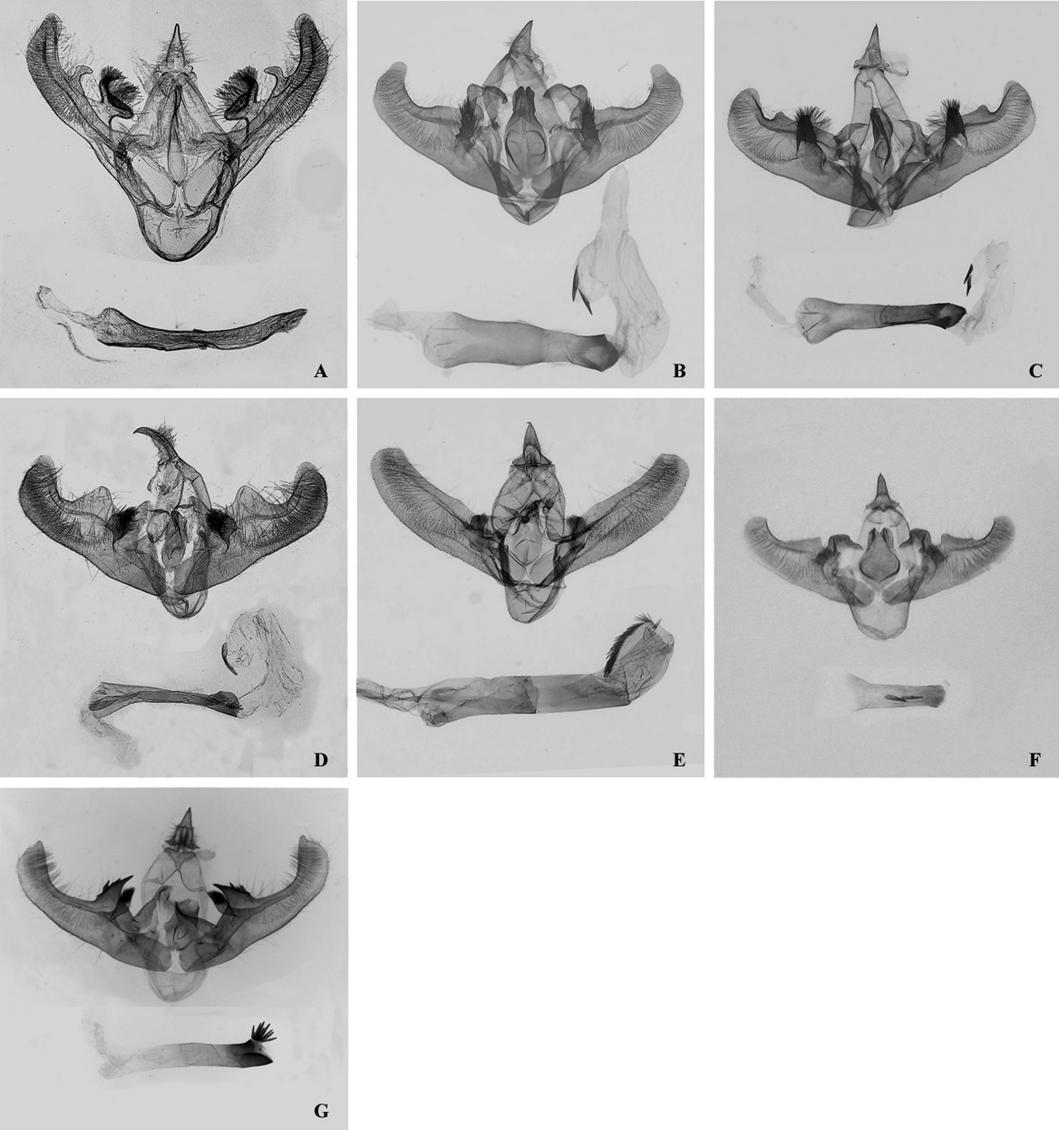
Male genitalia of *Orthobrachia* species. **A** *Orthobrachia latifasciata* (Moore, 1888), Lectotype **B** *Orthobrachia flavidior* (Hampson, 1898) **C** *Orthobrachia tenebrosa* Yazaki, 1992, paratype **D** *Orthobrachia owadai* Yazaki, 1992, holotype **E** *Orthobrachia simpliciata* Yazaki, 2002, paratype **F** *Orthobrachia maoershanensis* Huang, Xin & Wang, 2003, holotype **G** *Orthobrachia hirowatarii* Huang, Su & Stüning, sp. n., holotype.

**Figure 5.**
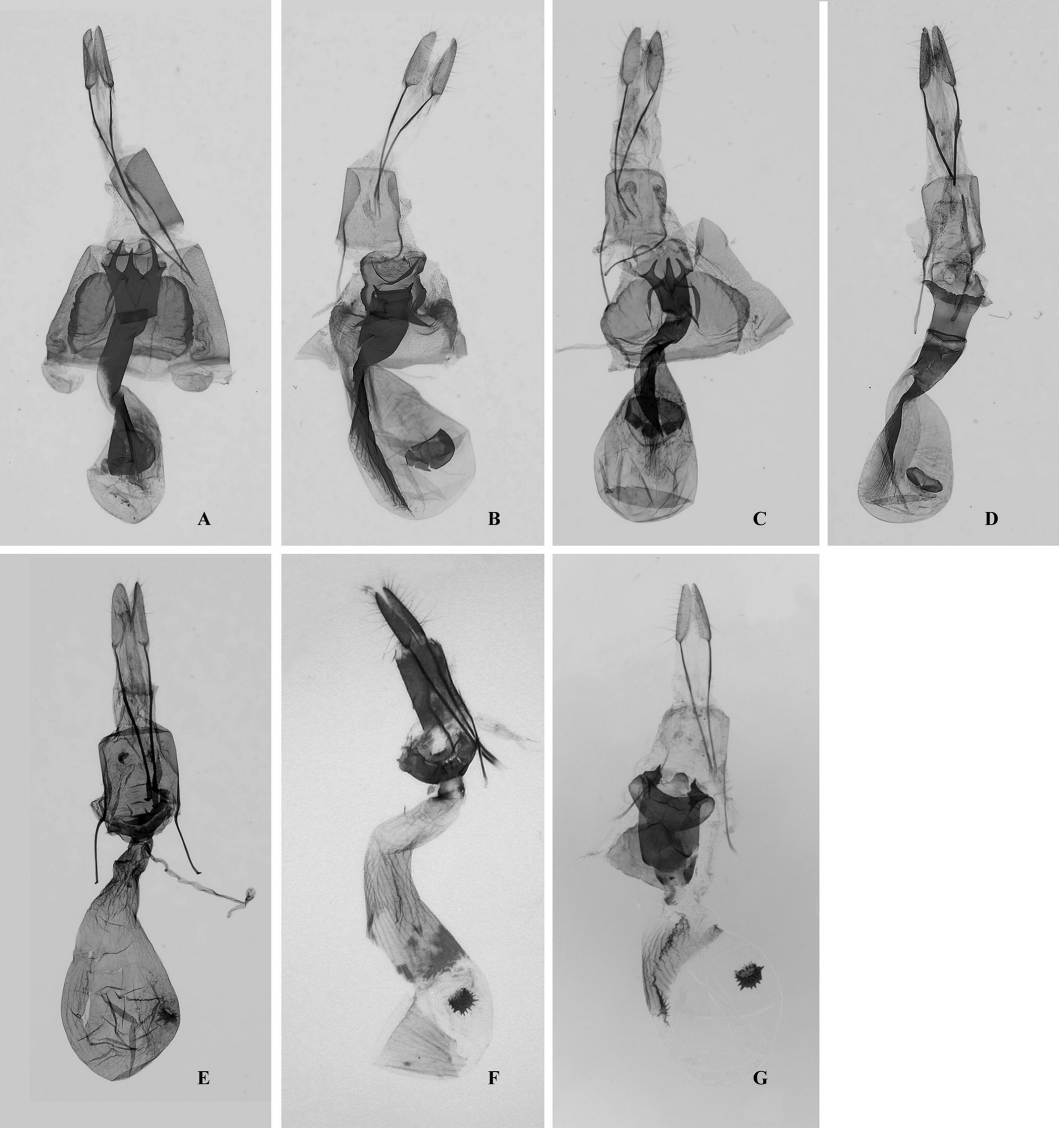
Female genitalia of *Orthobrachia* species. **A** *Orthobrachia latifasciata* (Moore, 1888) **B** *Orthobrachia flavidior* (Hampson, 1898) **C** *Orthobrachia tenebrosa* Yazaki, 1992, paratype **D** *Orthobrachia owadai* Yazaki, 1992, paratype **E** *Orthobrachia simpliciata* Yazaki, 2002, paratype **F** *Orthobrachia maoershanensis* Huang, Xin & Wang, 2003, paratype (bursa copulatrix ripped on left side) **G** *Orthobrachia hirowatarii* Huang, Su & Stüning, sp. n., paratype (bursa thinly membranous, margins only faintly visible).

#### Material examined.

INDIA, 1♂, Holotype, West Sikkim, Choka, 3050 m, 23-24.IX.1983, M. Owada leg., NSMT. 1♂, Indien, W. Bengal, 2400 m, Darjeeling, Tigerhill, 10-12.VII.1986, leg. W. Thomas. Gen. prep. no. 2305-DS. ZFMK.

#### Biology.

The two adults known were flying in Juli and September, at elevations between 2400 and 3050 m.

#### Distribution.

N.E. India, Sikkim.

#### Remarks.

This species has so far been observed near the type locality only.

### Orthobrachia tenebrosa

Taxon classificationAnimaliaLepidopteraGeometridae

Yazaki, 1992

Figures 3A , 4C , 5C

Orthobrachia tenebrosa Yazaki, 1992, Tinea, 13 (Suppl. 2): 23. Type locality: Gandaki Parbat District, Nepal.

#### Diagnosis.

This species is similar to *Orthobrachia latifasciata*,﻿ *Orthobrachia flavidior* and *Orthobrachia owadai*, especially similar to *latifasciata*, which has very similar transverse lines and also a large, dark brown apical patch in the hindwing, but can be distinguished easily from all three species by the dark brown medial area of the hindwing which are white or greyish-white in the other three. Moreover, the postmedian line is situated more distally. In the forewing, the broader median area is more strongly suffused with greyish-brown (see also key to species). In the male genitalia, length and width of the uncus are intermediate between *latifasciata* and *flavidior*. The valva bears a small triangular, apically rounded costal process arising from beyond the middle, and the saccular lobe is longer, extending more dorsally beyond dorsal margin of the valva, compared to *flavidior*. The aedeagus is more slenderly built and shorter, two cornuti are present on vesica, but smaller in size. There is no external bunch of spines like in *owadai*. The female genitalia are very similar to those of *latifasciata*, but the antrum is smaller, with the lateral pair of spines of equal length of the central one. In *latifasciata*, the lateral spines are longer.

#### Material examined.

NEPAL: 1♂, Holotype, Gandaki Parbat District, Ghorapani, Deolari, 2800 m, 15.X.1981, M. Owada leg., NSMT; Paratypes, 5♂♂, Same data as holotype. INDIA: 1♀, Western Bengal, Tiger Hill, 2573 m, 30.IX-5.X.1986, F. Aulombard & J. Plante leg., BMNH.

#### Biology.

The adults are flying in September and October in high elevations between 2500 and 2800 m.

#### Distribution.

Nepal, NE. India

#### Remarks.

The female from Sikkim, designated as paratype by Yazaki (1992), is the only female known so far. We figure its genitalia here, provided by Mr. K. Yazaki.

### Orthobrachia simpliciata

Taxon classificationAnimaliaLepidopteraGeometridae

Yazaki, 2002

Figures 3C–D , 4E , 5E

Orthobrachia simpliciata Yazaki, 2002, Tinea, 17 (1): 32. Type locality: Taiwan, China.

#### Diagnosis.

This species is characterized in appearance by rather long antennal rami in the male, less yellowish (rather creamy white) wings, especially in the distal third of the hindwing, somewhat ill-defined transverse fasciae, and in the hindwing-pattern consisting of an incomplete postmedial line only. In the male genitalia the valva is simple, almost not curved dorsad at apex and any costal ornamentation, such as small processes, present in *latifasciata* and *tenebrosa*,﻿ or the large dorsal expansions in *flavidior* and *owadai*, ﻿are absent. The saccular process is small and situated close to the base of valva. The aedeagus is unique in having a large row of small cornuti on vesica, together with a terminal, stronger and straight cornutus. Female genitalia are very different compared to *latifasciata*, *flavidior*, *tenebrosa* and *owadai* (“*latifasciata*-group”). The distinctive differences unite *simpliciata* with *maoershanensis* and *hirowatarii* (the *simpliciata*-group): with an arcuate, narrow lamella antevaginalis instead of a spined antrum, without long sclerotized bands and with an asymmetric, stellate signum. *Orthobrachia simpliciata* exhibits the most plesiomorphic characters in the female, but also in the male genitalia.

#### Material examined.

TAIWAN: 1♂, Holotype, Taichung, Mt. Anmashan, 2350 m altitude, 1.IV.1996, H.R. Tzuoo leg., NMNS; Paratypes 1♂2♀♀, same data as holotype, 1♂ same locality as holotype except for 9.III.1996, Fu leg., NSMT.

#### Biology.

The species was flying in spring at the type-locality.

#### Distribution.

China (Taiwan).

#### Remarks.

This species is endemic to Taiwan.

### Orthobrachia maoershanensis

Taxon classificationAnimaliaLepidopteraGeometridae

Huang, Xin & Wang, 2003

Figures 3E–F , 4F , 5F

Orthobrachia maoershanensis Huang, Xin & Wang, 2003, Tinea, 17 (5): 229. Type locality: Guangxi, China.

#### Diagnosis.

The species is externally very similar to the new species described below, to *Orthobrachia simpliciata* and also to *Orthobrachia tenebrosa*,﻿ but can be distinguished from all by the following characters: The antemedial and postmedial lines of the forewing are distinctly waved in *tenebrosa*, in *maoershanensis* almost straight, only the postmedial is slightly curved inward near costa of forewing, straight in the new species. From *Orthobrachia simpliciata*, it can easily be separated mainly by the differences in the hind wing pattern described above. Further distinguishing features see next species. The male genitalia of all three species have clear specific characters, the female genitalia as well, the latter uniting *simpliciata*, *maoershanensis* and *hirowatarii* sp. n. by a number of characters shared.

#### Material examined.

CHINA: 1♂, Holotype, Mao’ershan National Nature Reserve, 2000 m altitude, 25°54'N, 110°30'E, Primary forest, Xin’an County, Guangxi Province, China, 28.VI.2003, G.H. Huang leg., Gen. prep. no. HGH-SCAU_0011, SCAU; Paratypes 2♂♂7♀♀, same locality as in the holotype, 1600-2000 m, 28.VI-4.VII.2003, M. Wang and G.H. Huang leg., Gen. prep. no. HGH-SCAU_0013 (female), SCAU (1♂1♀ donated to Institute of Zoology, Chinese Academy of Sciences, Beijing (IZCAS)); 1♂, Huilongsi, Mao’ershan National Nature Reserve, 1489 m, 10.IX.2015, M. Wang leg., HUNAU. 1♀, N. Vietnam, Cha-pa, Mt. Fan-si-pan, 22°15’N 103°46’E, 1500-1800 m, 10.VI-6.VII.1994, lux, leg.V. Sinjaev & local coll., ZFMK.

#### Biology.

The species was collected in June-July and again in September in a primary forest near the top of a mountain, at elevations between 1500 and 2000 m.

#### Distribution.

China (Guangxi), Vietnam.

### Orthobrachia hirowatarii

Taxon classificationAnimaliaLepidopteraGeometridae

Huang, Su & Stüning sp. n.

http://zoobank.org/EB40BAE2-48B5-408D-94ED-E45F7D8AA338

Figures 3G–H , 4G , 5G

#### Diagnosis.

This new species is externally very similar to *Orthobrachia maoershanensis* but can be distinguished by the dark-brown ante- and postmedial lines, which are stronger and straight in *hirowatarii*, more delicate and the postmedial line curved inwards near costa in *maoershanensis*. The tornal dark brown patch is broader and shorter in the latter, reaching up to the middle of the medial band; the narrower patch of *hirowatarii* is longer and reaches almost back to the antemedial line. In the hind wing, the narrow, almost black postmedial line is almost straight between apex and tornus in *hirowatarii*, while it is evenly curved between anterior and posterior margin distinctly basad of apex and tornus in *maoershanensis*. The valve in the male genitalia is longer and narrower with two processes near the base in *hirowatarii*, a multi-dentate costal process and an arm-shaped, angled, apically densely setose saccular process. The costal process in *maoershanensis* is short and apically rounded, the saccular process thumb-like, with shorter setae at tip. In addition, the latter has a broad, basal costal process, similar to that found in *flavidior*. The aedeagus is short, stout, with two cornuti in *maoershanensis*, while *hirowatarii* has a longer, narrow aedeagus with a bunch of external spines at the border between shaft and vesica. The female genitalia are also clearly separable, though both (and *simpliciata*, too) have a similar, asymmetric, stellate signum (very small in *simpliciata*). *Hirowatarii* has a large, rather quadrate, strongly sclerotized antrum, with a transverse, semicircular, broad lamella antevaginalis distally, decorated with a pair of lateral spines. In *maoershanensis*, the quadrate part is absent, the semicircular part more delicate and the spines smaller. Moreover, *maoershanensis* has a much longer, fluted ductus bursae and the bursa is smaller (in our Fig. 5G with a damage on the left side).

#### Description.

♂ Expanse 27–30 mm, length of forewing 13–15 mm.

Head. Antenna bipectinate to three-fourths, rami arising from the proximal one third of flagellomers, apical 15–16 segments not pectinated. Frons narrow, smooth-scaled, lower half with yellow, upper half with greyish-brown scales. Vertex with larger, creamy white scales. Palps narrow, short, scaled light greyish-brown. Thorax. Patagia greyish-brown, tegulae creamy white, thorax subdorsally with two longitudinal, dark brown lines (which are continued on the abdomen). Forewing ground colour creamy white, with transverse striation of dark brown and orange, scales of the same colours also accompany the veins. There is a dark brown patch at tornus, extended along posterior margin, reaching almost the antemedial line. The latter line strong, dark brown, straight, shortly curved basad and broadening near costa; postmedial line straight from tornus to 1/4 of costal margin; there is a small, semicircular loop between veins R_5_ and M_1_ at termen (also present in *maoershanensis* and *simpliciata*); cilia dark brown at posterior half of termen, creamy white between the dark end of the veins in anterior half. Hindwing rounded; ground colour similar to forewing, with broad, greyish-brown medial area. Thin blackish-brown postmedial line straight between tornus and apex, discal cell with a small black spot. Cilia brown, apart from those in the spaces M_1_-M_3,_ M_3_-CuA_1_ and CuA_1_- CuA_2_ which are creamy white.

♀ Expanse 30–34 mm, length of forewing 14–16 mm, antenna filiform. The ground colour of the wings and the pattern elements are very similar to the male.

Male genitalia: Uncus rather broad and short, beak-shaped. Gnathos consisting of weak, flattened lateral arms only, not fused in the middle. Valva long and narrow, with the distal end of costa projecting above the valve surface. Costa with a large, multi-dentate process just basally of the middle, and saccular process with a narrow basal arm and a broader, globular, apically densely setose distal parts. Juxta a large, broad, somewhat elongated plate. Saccus broad and rounded, flattened at base. Aedeagus slender with a bunch of well-developed cornuti, arising externally at the border between shaft and vesical, the latter without cornuti.

Female genitalia: Papillae anales elongate, apophyses long; a. anteriores about 3/5 the length of a. posteriores; lamella antevaginalis well sclerotized, semicircular, with a pair of spines bilaterally, united with a large, nearly quadrate, well sclerotized antrum; bursa copulatrix thinly membranous, pear-shaped (not clearly visible in our figure), distal part sclerotized, fluted, containing a wrinkled band; ductus bursae very short; signum rounded, margins and internal surface covered with spines.

#### Holotype.

♂, China: Sichuan, Yingjing County, Longcanggou Town, 1420 m, 10.VIII.2015, light trap, G.H. Huang leg., Gen. prep. no. HGH-HUNAU_0165 deposited in HUNAU.

#### Paratypes.

5♂♂8♀♀, same locality as holotype, but 09-11.VIII.2015, G.H. Huang, T. Hirowatari, T.T. Yu and M. Wang leg., Gen. prep. no. HGH-HUNAU_0168 (female), deposited in HUNAU and SCAU. 1♂, N.Thailand, Changwat Chiang Mai, 23 km NW Sop Kha, 1 km E Kop Dong, 1650 m, 29.I.2000, leg. Márton Hreblay. Gen. prep. no. 2304-DS, ZFMK

#### Etymology.

The specific epithet is in honour of Prof. Toshiya Hirowatari, who was the supervisor of the first author for Ph.D. Course in Osaka Prefecture University.

#### Biology.

The adults fly in August in Sichuan, in January in Thailand, at elevations between 1400 and 1700 m. The host of larvae is unknown.

#### Distribution.

China (Sichuan), Thailand.

#### Remarks.

This new species was collected at a small village in the forest, with a light-trap inside a house, with artificial vegetation around it. Therefore the habitat seems to be quite different from that of other *Orthobrachia* species. E. g., *Orthobrachia maoershanensis* originates from environment with natural vegetation in the core zone of the Nature Reserve.

## Conclusions and discussion

The systematic relationship of the genus *Orthobrachia* has been questioned for a long time, as explained in the introduction chapter. We are convinced that it is a member of the tribe Baptini (sensu Holloway (1994)) indicated by the following characters: 1) vein R_2_ in the forewing arising from a common stalk with R_3_-R_5_; 2) fovea in forewing absent; 3) transverse comb of setae on sternite 3 absent; 4) valves elongate, more or less parallel-sided, rounded at apex, with a broad, immaculate costal zone, often with a marginal process or angle, an elongate field of setae, often with peg-like, short, broad setae (the latter not present in *Orthobrachia*, but also not present in a number of other genera of Baptini, sensu Holloway (1994)); 5) gnathos weak or absent (in *Orthobrachia* separated lateral arms present only). All or most of these characters are present in the genus *Orthobrachia*. Related genera in the Baptini are *Platycerota* Hampson, 1893 (= *Crypsicometa* Warren, 1894) and *Heterostegania* Warren, 1893.

*Orthobrachia* species are distributed from NW India (Kashmir) to Zhejiang and Taiwan (E. China), S. China, N. Vietnam and N. Thailand (the China-Himalayan animal area, as considered by Huang et al. 2010). *Orthobrachia latifasciata* and *Orthobrachia flavidior* are the most widespread species, *latifasciata* occurring from Kashmir to Western Central China, *flavidior* from C. Nepal to E. China (Zhejiang), the latter also being the most abundant of all species. Most other species seem to be rare or are at least rarely collected. *Orthobrachia tenebrosa* is only known from a few specimens from C. Nepal and Sikkim, NE. India, *Orthobrachia owadai* from the latter region only. Species of the *simpliciata*-group are only distributed in E. and SE. China, including the more northern Sichuan region. *Orthobrachia simpliciata* is endemic to Taiwan, and also the recently described *Orthobrachia maoershanensis* and *Orthobrachia hirowatarii*,﻿ described herein, were considered to be very local species firstly, but the discovery of both species in N. Vietnam and N. Thailand, respectively, proved them to be more widespread. More collecting at appropriate places (i.e. natural forests at elevations between 1500 and 3000 m a.s.l.) will probably reveal even more localities where these rare species occur. Considering the fact, that no *Orthobrachia* species are known at all to us from N. Myanmar and N. Laos, we also believe that the discovery of further new species is possible.

## Supplementary Material

XML Treatment for Orthobrachia

XML Treatment for Orthobrachia latifasciata

XML Treatment for Orthobrachia flavidior

XML Treatment for Orthobrachia owadai

XML Treatment for Orthobrachia tenebrosa

XML Treatment for Orthobrachia simpliciata

XML Treatment for Orthobrachia maoershanensis

XML Treatment for Orthobrachia hirowatarii
